# Product News

**DOI:** 10.1155/S1463924680000491

**Published:** 1980


### Product News

### Pressure regulators

Tescom Corporation have extended their 44-2200 series of pressure regulators to meet the requirements for fluids regulation in semiconductor and electrical component manufacturing processes. High purity, corrosive and poisonous fluids at pressures up to 3000 psig can be accurately regulated by the stainless steel model 44-2260. All parts which are exposed to the flowing media are constructed of 316 stainless steel with only one soft seal which is a Teflon seat. Problems associated with soft seals are overcome by a metal to metal diaphragm seal beween the regulators body and bonnet. A specially designed convoluted stainless steel diaphragm provides stable regulation of delivery pressure. Four outlet ranges are available which suit different manufacturing cell requirements. The regulator has an optional gas-sealed bonnet which helps prevent contamination of the work area in the event of a diaphragm failure or overload.
*Techmation Ltd, 58 Edgware Way, Edgware, Middx, UK.*

### Density measuring instrument

Laboratory Impex have introduced the Berthold density measuring instrument, the LB379, for continuous solution density measurement. The stainless steel instrument operates on a scintillation counting basis and has the advantage of giving continuous measurement with contact with the medium involved. It can be used to determine liquid concentration in a wide range of situations, including sugar refining, milk processing, acids, lyes and salt solutions. A special lining for aggressive or abrasive media is available. The sample density signal output from the detector is both linearised and temperature compensated; the reading is displayed on the amplifier and readout unit used with the detector.
*Laboratory Impex Ltd, Lion Road, Twickenham, Middx, UK.*

### Servo-recorder

Radiometer has announced the REC80 Servo-Graph, a servo recorder for general purpose applications in the analytical laboratory. It is driven by a stepping motor and has reversible, calibrated chart feed between 1s/cm and 2h/cm. Response time of the servo driven pen is less than 0.6 s. It is suitable for high-sensitivity analogue recording down to 4 uV/cm and for pH, pX and redox recordings. In conjunction with their titration system it will perform conventional, stepped and derivative titration curve recording. The recorder will register the potential versus time curves when performing trace metal analysis with their ion scanning system and when connected to their conductivity meter, a conductometric curve recording is obtained.
*Radiometer A/S, Emdrupvej 72, DK-2400, Copenhagen NV, Denmark.*

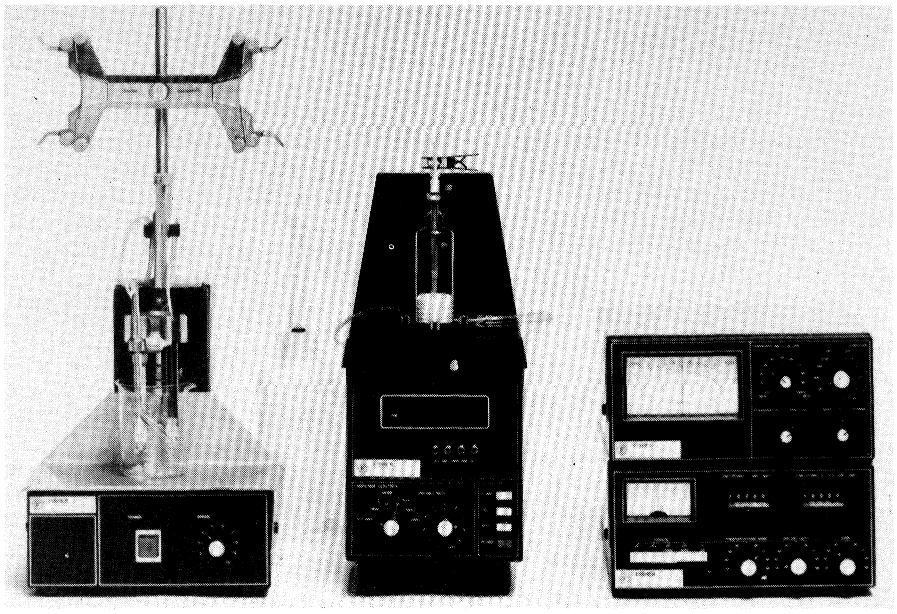

*The Fisher Titrimeter II AEP titration system*

### Clinical thermometer

The Digitron 4701 and 4702 electronic clinical thermometers from Electroplan are instruments designed to replace mercury-in-glass type thermometers. The probes consist of precision thermistors in flexible plastic sheaths that may be chemically sterilised. The 3-digit LED display gives a resolution of 0.1°C with an accuracy of ± 0.1°C over the range 33-43°C.

A wide variety of probes are available for different applications such as surface measurements as well as an optional rechargeable battery pack
*Electroplan Ltd, PO Box 19, Orchard Road, Royston, Herts SG8 5HH, UK.*

### UV-visible spectrophotometers

Two microcomputer controlled scanning spectrophotometers, the DMS 80 and the DMS 90 have been introduced by Varian. Both instruments are compact, rugged and have been designed for routine and non-routine analytical and research laboratories. The software for the built-in Intel 8085A microcomputer has been programmed specifically with the operator in mind. Key features include simple operation parameter selection, auto-zeroing, a wide range of photometric modes, single or double beam versatility, sealed high energy optics and external computer compatibility.
*Varian AG, Steinhauserstrasse, CH-6300 Zug, Switzerland.*

### Continous flow analysis equipment

ChemLab Instruments have published a catalogue for their complete range of automatic continuous flow analysis equipment. It contains information on all the equipment available, including the latest designs of high temperature and dialysis baths, CAAII type analytical cartridges and peristaltic pumps with 'stand-by' facility.

A brief description is given of the two systems which have evolved since the concept of flow analysis was first introduced and of the methodologies evolved since the concept of flow analysis was first introduced, and of the methodologies which can be carried out on them.
*ChemLab Instruments Ltd, Hornminster House, 129 Upminster Road, Hornchurch, UK.*

### Titration system

The Titrimeter II AEP System from Fisher is a semi-automatic single-sample titrating system. It can operate in both fixed-endpoint and endpoint-seeking modes and can handle aqueous and non-aqueous samples. The system can titrate to all equivalence points within limits set by the user, calculate and printout all endpoints and provide a volume derivative recording of results. In both modes, endpoint approaches are proportionally controlled, with titrant delivery rate continually decreasing as the endpoint is approached. Limits for endpoint seeking titrations and fixed endpoints are set directly on digital switches. Microprocessor control automatically delivers titrant and the dispensed titrant volume is continuously displayed on a LED readout.
*Fisher Scientific Co, 711 Forbes Ave, Pittsburgh, PA 15219, USA.*

### Automatic burette

Radiometer has announced a new automatic burette, the ABU80 Autoburette, for high-precison, computerised titrant delivery. The two-in-one burette assemblies are available in the versions 25 ml/2.5ml, 10 ml/1.0 ml and 2.5 ml/0.25 ml and are seated within the housing for maximum protection. The autoburette can be set to manual or fully automatic operation.

The chemically resistant stopcock is grease-free. Eleven titration speeds between 5 and 160% of total burette vol/min assure optimal titration conditions. Titrant bottles are available in both clear and amber glass. A special flush programme at start-up ensure that all tubings are filled with fresh titrant and that all air bubbles are expelled.

*Radiometer A/S, Emdrupvej 72, DK-2400, Copenhagen NV, Denmark.*

### Chromatography data stations

Perkin-Elmer have introduced new versions of their Sigma 10 data station, the Sigma 10B/2 and Sigma 10B/4 which offer greater flexibility of configuration than the previous systems.

The Sigma 10B/4 can simultaneously support data collection and reduction from 4 chromatographs, data storage and retrieval from a magnetic tape cassette and an RS232C communications port. The latter facility when combined with the BASIC II programmability allows twoway data transfer under synchronised control to a mainframe computer.

The Sigma 10B/2 supports data collection from 2 chromatographs in addition to the other features of the Sigma 10B/4. Both models when used with the AS-100 liquid autosampler can provide total automated control via a BCD reader and BASIC II.

*Perkin-Elmer Ltd, Post Office Lane, Beaconsfield, Bucks HP9 1QA, UK.*

### Coal analyser

Fisher Scientific has announced the introduction of the Model 490 Coal analyser. This instrument analyses a single sample for % moisture, volatiles, ash and fixed carbon. The analyser can process up to 80 samples in a day's run and the results are claimed to meet or exceed repeatability and reproducibility requirements of ASTM method D3172, the proximate analysis of coal and coke.

The analyser contains oven, furnace, desiccator, and microprocessor modules in a self-contained unit. It also comes equipped with an interface for the Mettler A-30 high-speed balance for automatic sample weight entry. The analyser can be used for applications in the food and chemical industries.

*Fisher Scientific Co, 711 Forbes Ave, Pittsburgh, Pa 15219, USA.*

### Automatic titration system

Radiometer has announced a new 880 Kappa number titration system for the determination of the Kappa number of pulp. After transferring the pulp sample and sulphuric acid to the 1 litre reaction vessel and depression of the start button, the analysis is performed automatically. The necessary reagents are added at the required time intervals, and the final redox titration with thiosulphate is potentiometrically controlled and stopped precisely at the end-point. The volume of thiosulphate consumed is indicated on a 4-digit LED display. The repeatability of the 'Kappa number' determination is 1%.

*Radiometer A/S, Emdruvej 72, DK-2400 Copenhagen NV, Denmark.*

### Radioimmunassay standards

Advanced Laboratory Techniques has available a complete range of standards for the radioimmunoassay of T4, T3, h-TSH, alpha fetoprotein, prolactin and digoxin. These standards are supplied either in liquid or lyophilised form across a range suitable to run at least five complete standard curves, and are stable either deep-frozen or at 4°C (lyophilised) for a minimum of six months.

*Advanced Laboratory Techniques, 1 Clanricarde Gardens, Tunbridge Wells, Kent, UK.*

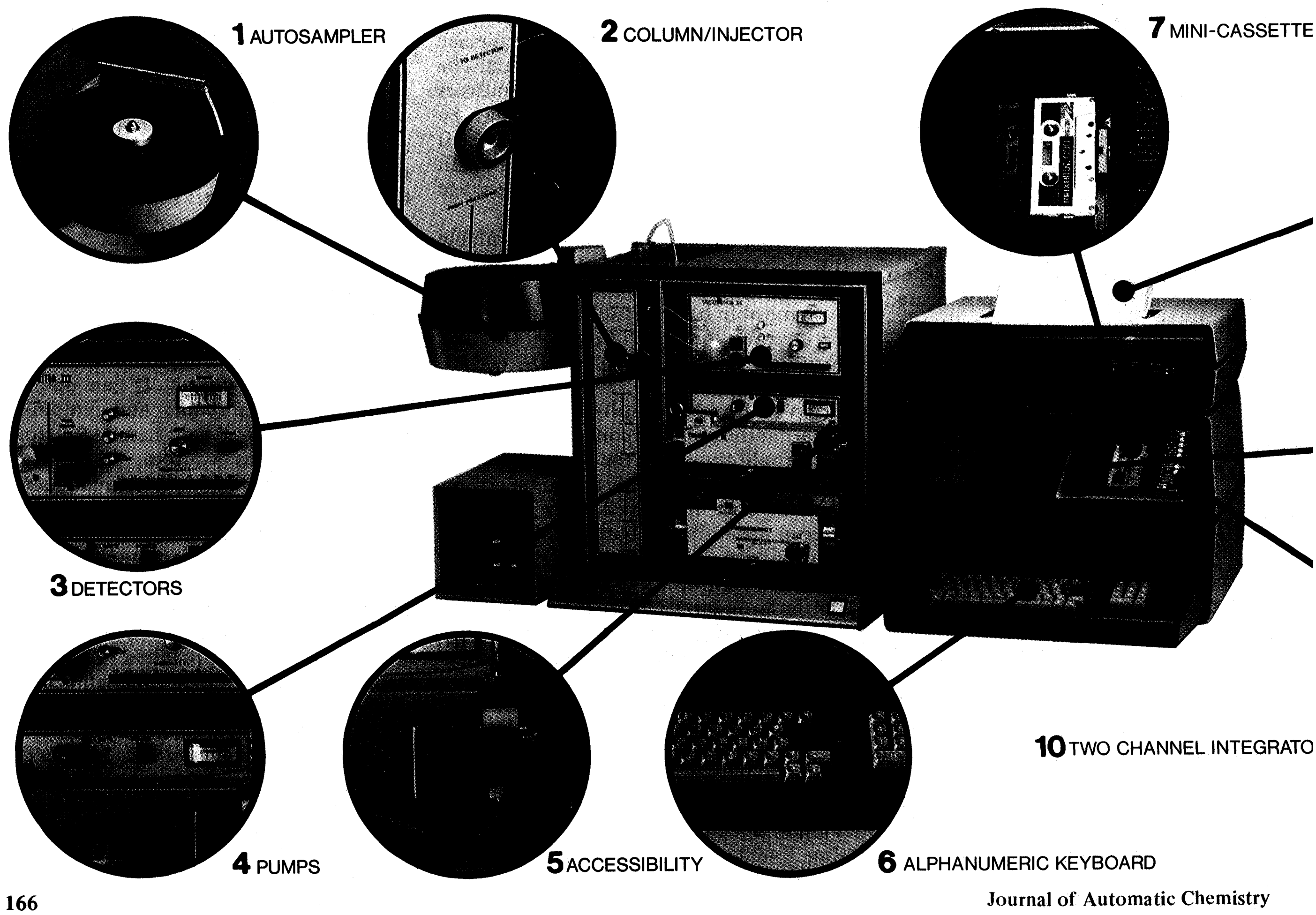

### Diluter/dispenser

V A Howe and Co is now marketing a microprocessor controlled diluter/dispenser, the Microlab M. This instrument is designed to fulfil all dispensing and dilution applications in clinical laboratories. The unit incorporates a high resolution, high performance stepper motor with synchronised motor driven valve block. An integrated standard EIA RS 232 C interface is available. It can accommodate all standard Hamilton precision syringes from 50 µl to 25 ml. The instrument is operated by an external control unit with an integrated keyboard and display. A total of 99 programs including 384 individual volume steps or functions can be stored. The algebraic input allows the instrument to be programmed in the diluter mode with an air bubble between the sample and the diluent.

*V A Howe & Co Ltd, 88 Peterborough Road, London SW6, UK.*

### Modular HPLC systems with CRT controller

Altex have introduced two new HPLC systems, models 324 and 334, each has a CRT controller which allows easier operation and simplified methods development. More efficient software, a multifield display and larger memory capacity provides total system monitoring. Operator controls system functions from an easy-to-read keyboard, and programs can be designed for unattended analyses and chromatographic data processing. Digital tape cassette is optional and can be used to build a methods library for error-free runs with minimal system set-up time.

Both instruments offer pulse-free solvent delivery and may be operated in either isocratic, gradient or ternary mode with optimal components. The Modules capable of stand-alone operation. The model 324 operates at pressures up to 10,000 psi and the model 334 operates at pressures up to 6,000 psi.

*Altex Scientific Inc, 1780 Fourth Street, Berkeley, CA 94710, USA.*

### Automated data handling

Technicon Industrial Systems have announced the introduction of their AutoAnalyzer IIC system for fully-automated data handling and instrument control. The system consists of three modules, microprocessor, an automatic valve and an input/output terminal.

The new system is an addition to the existing continuous flow analytical systems, the basic systems utilised to automate over 500 different wet chemical analyses. It extends automation beyond the wet chemical analysis, providing automated start-up and shut-down of the instrument, and full automation of data-handling, corrected and real-time, using the latest peak-picking technology with corrections for drift, sensitivity and carryover. Continuous-flow functions, such as peak shapes, are routinely monitored and flagged, if abnormal or outside limits previously set in the programme. The output is provided in final report form. It is available with up to four-channel capability.

*Technicon Industrial Systems, Tarrytown, New York 10591, USA.*

### Translation stages and linear micro-actuators

Unimatic Engineers have introduced their Micro-Control UT range of translation stages, with linear travel from 1 to 125 mm. These stages can accept loads of up to 25 kg and develop positioning speeds up to 2 mm/sec, with a positioning resolution from 0.1 u. The UT-100 units are modular and can be used with linear, xy or multi-axis systems. They are primarily used in physics/optics research, in the construction of optical instruments and in the micro-electronics industry.

*Unimatic Engineers Ltd, Granville Road Works, 122 Granville Rd, Cricklewood, London NW2, UK.*

**Ten Good Reasons for Investing in LDC's Successful CCM-Based System for HPLC...**

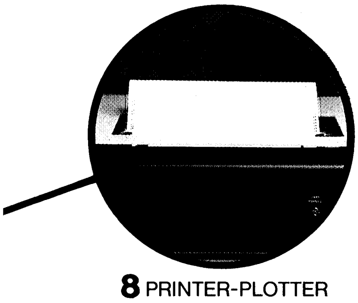

**8** PRINTER-PLOTTER

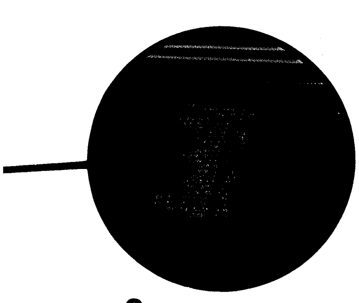

**9** VIDEO DISPLAY

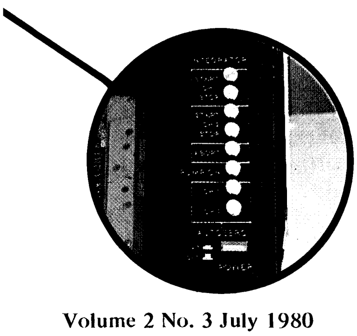

**1** Automatic sample injection with search facility
**2** Easy access to column compartment for maximum flexibility
**3** Wide choice of detection options available:
UV III Monitor fixed wavelength UV detector for sensitivity (0.002 AUFS)
spectroMonitor III variable wavelength UV detector for versatility (190-350 nm)
fluoroMonitor III fluorescence detector with $10^{-18}$ mole sensitivity
refractoMonitor III refractive index detector for universal applications
**4** Reliable pumps—the product of 30 years experience in laboratory pump technology
**5** Easy access for routine maintenance of both columns and detectors
**6** Full alphanumeric keyboard for easy communication
**7** Cassette system for data and programme storage
**8** Two-pen thermal printer-plotter provides hard copy of complete chromatogram with associated digital report
**9** Large shielded visual display in plain language instead of mneumonic codes. Core resident BASIC for true computer capability.
**10** Simultaneous dual channel integration and full data processing

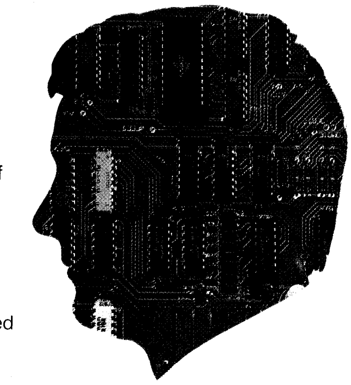

Yes! The versatility of LDC's Analyst coupled with the power of the CCM (Chromatography Control Module) combine to give
—**maximum user control**
—**maximum credibility of results**
—**maximum versatility in chromatography**
—**maximum use of chemist's skill and time**
Ask now for detailed specifications to streamline your HPLC laboratory with the CCM-based system—today's most advanced generation of analytical control systems.

**LABORATORY DATA CONTROL** BRAINPOWER FOR CHROMATOGRAPHY.

*LDC is a division of Milton Roy Company, Florida: one of the World's leading manufacturers of laboratory instrumentation.*
**Laboratory Data Control, Shannon International Airport, Ireland. Tel: 061-61266 Telex: 6227.**
**U.S.A.:** P.O. Box 10235, Riviera Beach, Florida 33404. Tel: 305/844-5241 Telex: 513479. **U.K.:** Milton Roy House, High Street, Stone, Staffs. ST15 8AR. Tel: 0785 83 3542 Telex: 36623.
**West Germany:** Milton Roy GmbH., 6467 Hasselroth/2, Jahnstrasse 22-24. Tel: 6055 33 66 Telex: 4184357. 004

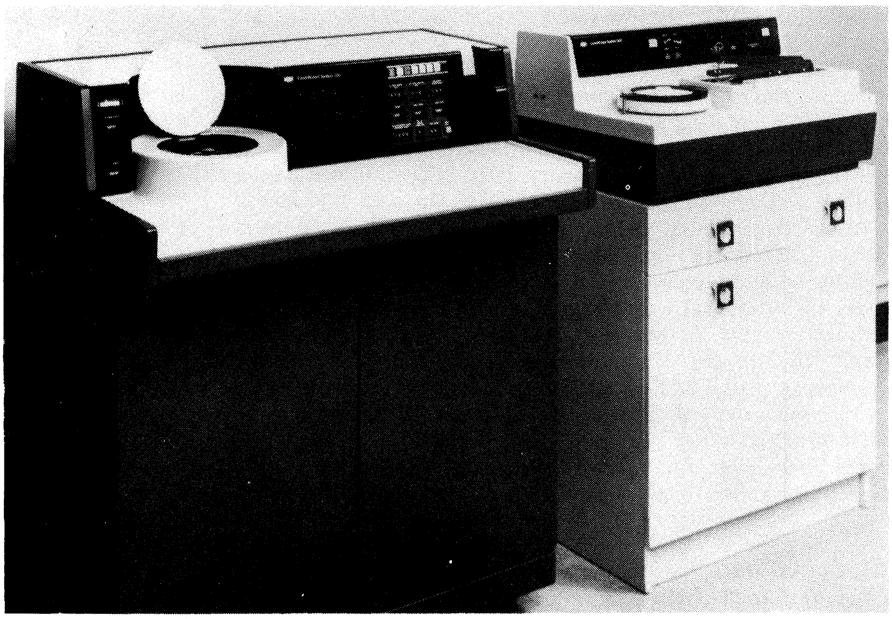

*The CentrifiChem System 500 Centrifugal blood analyser from Union Carbide*

### Analytical monitors

Breda Scientific has announced a new series of analytical monitors. The main application of this instrument is to examine one constituent in a liquid contained in a vessel, flowing in a pipe-line or an open channel. It is equipped with an on-line sampling system, introducing selectively either sample, standard or blank to the analytical part which uses a flow through optical detector. The integrated time controlled sequencing allows for automatic drift correction and self calibration of the system. A range of specific industrial requirements can be handled covering both high and low range concentrations, from 0-50 ppb up to 98%.

*Breda Scientific, Cenco Instrumenten BV, Konijnenberg 40-4825, BD Breda, PO Box 3336-4800 DH Breda, The Netherlands.*

### Centrifugal blood analyser

The Medical Products Division of Union Carbide UK has recently introduced a centrifugal blood analyser, the CentrifiChem System 500. The principle of the system is the use of centrifugal force to mix the patient's sample with the corresponding biochemical reagent to give rapid diagnostic results. Over 1,000 blood samples can be assayed per day on the system which can be operated by a single technician.

Small quantities of blood are required from the patient in order for tests to be carried out and the tests require 50% less than normal of the biochemicals.

Test results are by paper print-out or shown on a screen by linking the system to an intelligent computer VDU unit or desk calculator. The system can also be programmed to operate with a full data handling mini-computer package, which enables all the results to be collated and printed out automatically without manual transcription. Automatic quality control review programmes can be included, together with 'state of the art' data analysis and calculation facilities.

*Union Carbide UK Ltd, 8 Grafton Street, London W1A 2LR, UK.*

### GC Engineering Service

Pye Unicam has formed a new Projects Group to provide advice and assistance for analytical and industrial laboratories that need improved gas chromatography facilities. They aim to combine well-tried techniques with up-to-date technology to solve special problems, using purpose-built packages based on their GCD series 204 and series 304 range of laboratory gas chromatographs, solving individual customers needs economically and efficiently.

The options available include automatic sample injection, column switching with facilities for back-flushing and heart-cutting, automatic sequencing and data handling with microprocessors or interfacing with mainframe computors, together with a comprehensive range of columns and detectors.

*Pye Unicam Ltd, York St, Cambridge CB1 2PX, UK.*

### Gamma counter

The 'Micromedic' model 4/200 automatic gamma counter is now available from ChemLab Instruments. This model is a bench-top gamma counter with four detectors counting four tubes simultaneously. Processing is performed in the rack. Counting efficiency is claimed to be better than 75%. Count times range from 0.1 – 25.0 minutes. The throughput rate is 200 tubes per hour, based on a 1-minute counting time. A Model 4/600 which has a throughput rate of 600 tubes per hour is also available.

An integral microprocessor provides a digital display readout of counts for each tube, and also has a data reduction capability which can be programmed to reduce data automatically. The programme, designed specifically for chemical laboratories will accommodate most RIA methods and also allows users to insert their own parameters.

*ChemLab Instruments Ltd, Hornminster House, 129 Upminster Road, Hornchurch, RM11 3XJ, UK.*

### Monochromator

Rofin has announced the introduction of a monochromator which produces a scanning output which enables optical spectra to be displayed on an oscilloscope. The basic unit generates a repeatedly swept spectral output and a trigger pulse for an oscilloscope. The detector option enables the user to display the optical spectra directly on a standard oscilloscope. Wavelength information may be obtained using either the oscilloscope graticule scale, or an accessory which gives marker pulses at 10 nm intervals. The wavelength marker accessory also provides a single large marker pulse which can be moved on the screen in steps of 1 nm by a digital thumbwheel switch.

The basic unit covers a spectral range of 400-700 nm although the full system is useable over the range 300-1100 nm. The wavelength accuracy is better than 1 nm and the bandpass with the narrowest slit is 2 nm.

*Rofin Ltd, Winslade House, Egham Hill, Egham, Surrey, UK.*

### Magnetic separation of antigen

Technicon has developed a method of separating the free antigen from the antigen-antibody complex by a magnet.

Specific antibodies are bound to a ferric oxide particle by a cyanogen bromide technique. Following incubation and the normal antibody-antigen reaction, the free and bound are separated by standing the solutions on a magnet, before removing the supernatant. The benefits of magnetic separation of bound and free antigen are realised in their fully automated RIA system, STAR. This system is microprocessor controlled, sampling at a rate of 60 per hour and reducing operator involvement. To perform the assay the operator simply loads the sampler and reagent and initiates the assay run by using the keyboard and visual display unit.

*Technicon Instruments Co Ltd, Evans House, Hamilton Close, Basingstoke, Hants RG21 2YE, UK.*

